# Maize (z*ea mays* l.) yield, and selected chemical properties of soils as influenced by lime and manure application in acid soils of Meket District, northeastern Ethiopia

**DOI:** 10.1371/journal.pone.0360551

**Published:** 2026-10-12

**Authors:** Endalkachew Fekadu, Yirbeb Woretaw, Mohammed Hussen

**Affiliations:** 1 Soil Resource and Watershed Management Department, Woldia University, Woldia, Ethiopia; 2 Plant Science Department, Woldia University, Woldia, Ethiopia; University of Salento: Universita del Salento, ITALY

## Abstract

Acid affected soils have been expanding in the Ethiopian highlands, and caused serious yield decline. Organic amendments and lime have been used to manage soil acidity. However, the rate and the right combinations have not been sufficiently documented for various crops and locations. This study aimed at investigating the effects of four manure rates (0, 4, 8, and 12 t ha^-1^), with three lime rates (0, 1.5 and 3 t ha^-1^ CaCO_3_) on maize yield, and selected soil chemical properties. The experiment was arranged in factorial combinations using randomized complete block design (RCBD) with three replications. The result showed that significant variations were obtained for yield, and yield components of maize where maximum grain yield (4087.0 kg ha^-1^) was harvested from the combination of 8 t manure ha^-1^ with 1.5 t lime ha^-1^. However, significantly (P < 0.01) highest stalk yield (11155.0 kg ha^-1^) and yield of dry aboveground biomass (14803.7 kg ha^-1^) were achieved from the integrations of 3 t lime with 12 t manure ha^-1^. The plots that received 12 t ha^-1^ manure only had the highest OC content (1.96%), with an increase of 54%. Following 3 t ha^-1^ lime plus 12 t ha^-1^ manure applications, the available P content was found to be significantly (P < 0.01) the highest (14.41 mg kg^-1^). The present experiment indicated that integrated application of 3 t ha^-1^ lime with 8 t ha^-1^ manure could help increase maize yield and profitability while maintaining fertility of soils in acid affected soils of Meket area and similar other locations.

## 1. Introduction

In most countries of Sub-Saharan Africa (SSA), which includes Ethiopia, soil fertility management is crucial given the region’s pervasive environmental degradation and ongoing poverty. Rain-fed agriculture makes up about 97% of the total agricultural area in SSA [1], which will soon continue to be the primary source of food production. In many African countries, crop productivity in the rain-fed systems is low. According to the 2023 crop data published by FAO [2], in Africa the average yields of maize was 2.1 t ha^-1^. Low yields are mostly the results of unsustainable soil management techniques, such as the removal of residues, minimal use of inputs, monocropping, soil acidity, and soil salinity [3].

Highland regions with mostly acid soils can be found in northwest, central Ethiopia, including Wolaita, Sidama, Gojam, Wollega, Arsi, and Bale. According to Regassa et al. [4], 21% of the total land area has soil pH values below 6. For sustainable crop production, such soils need to have their acidity managed. In Ethiopia’s northeastern highlands, soil acidity is one of the critical challenges affecting the production of acid-sensitive crops. Its effects on crop growth are associated with deficiency major nutrient (Ca, K, Mg, P and Mo), and the toxicity of ions of hydrogen (H), manganese (Mn), and aluminum (Al) to plant physiological processes [5]. Aluminum (Al) and Fe may shield organic carbon (OC) and protect microbial degradation, which in turn reduce OC availability in acid soils. Such acid ions also suppress enzymatic activities, and slow down nutrient cycling soils [6]. Low maize yield is also related to the result of phosphate being fixed and unavailable to plants when the pH of the soil is below 5 [7].

Fertilizers are essential for crop production and productivity, but if inorganic fertilizers are applied consistently in an unbalanced manner, the physical, chemical, and biological properties of soils deteriorate, ultimately affecting soil health and productivity [8]. The sustainability of crop production is adversely affected due to the changing health of the soil, the decrease in soil OC content, and the rise in micronutrient deficiencies [9].

Organic manures are known to have ample potential to enhance soil health, and significantly increase crop yields. Without adequate supply of organic matter OM), a soil cannot be deemed healthy. Many investigators [8,10,11] documented how important manure is for raising soil OC, available P, and total N while lowering soil acidity, either by itself or in conjunction with inorganic fertilizers. In the same way that organic manure improves soil structure, and water holding capacity, it also encourages root growth and boosts the activity of soil microflora and fauna, which enables plants to access the micronutrients [12].

The most widely used soil acidity management technique is use of agricultural liming material, which, when added to agricultural soil can minimize soil acidification and provide plant nutrients like Ca [13]. Liming soil has several advantageous effects, including improving soil structure, increasing the availability of P, Ca, Mg, and Mo, and neutralizing exchangeable Al [14]. Numerous studies have amply demonstrated the significance of liming acid soils. Lime decreases P fixation in acidic soils by lowering exchangeable Al^3+^, Fe^3+^, and Mn^4+^ levels. Furthermore, compared to other soil amendments like organic and inorganic materials, lime is known to have longer-lasting effects on acid soils.

Maize is one of the main cereal crops produced in most of the regions in Ethiopia. The high nutritional value of maize and the increasing need for the stalk as fuel for rural households account for some of the crop’s appeal in Ethiopia [15]. The productivity of maize in Amhara region is around 3.8 t ha^-1^ while the average national yield is 3.9 t ha^-1^, significantly less than the global average of 5.7 t ha^-1^ as reported by CSA [16]. In Ethiopian smallholder farms, soil acidification has been identified as one of the many factors limiting crop productivity [17]. Kumar et al. [18] indicated that addition of lime with manure and NPK improved maize productivity by more than three times. In Assosa, Ethiopia, lime application on acid soils had increased maize yields by improving the physical and chemical characteristics [19]. At Kwa Sadala and Mungushi, Tanzania soil pH was improved by 3.15 and 2.26%, respectively due to application of 7.8 t lime ha^-1^. Likewise, at Kwa Sadala, soil total N, OC, available P and exchangeable K^+^ increased by 16.67%, 18.13%, 20.95%, and 6.76%, respectively [20].

Soil acidity is among the many reasons why maize productivity is typically low in the Amhara region, particularly in Mekete District, despite the high potential of the crop for production. Soil erosion, leaching of basic cations, intensive and continuous land cultivation, and inadequate soil nutrient management practices are the observed causes of yield decline, and food security problems. Restoring soil nutrients and raising maize grain productivity in the study area thus require the identification of efficient Integrated Nutrient Management (INM) techniques. However, there is a scarcity of information in the study area to apply optimum combinations of lime and manure on maize, and soil chemical characteristics. As a result, a hypothesis was developed that states that applying varying rates of lime and cattle manure to acid soils significantly increase yield of maize. This experiment was designed with the objective of evaluating the effects of cattle manure with lime at different rates and combinations on maize yield, and selected soil chemical properties in Meket District, Ethiopia.

## 2. Materials and methods

### 2.1. Description of the study area

The study area was *Warkaye* farmers’ village, *Meket* District, North-wollo Zone, Ethiopia with an elevation of 2924 meters above sea level. The mean maximum and minimum temperatures in are 24 °C and 12 °C, respectively, while the average annual rainfall varies from 600 mm in the lowlands to 1120 mm in the highlands of the District [21]. The two most prevalent soil types in the research area are Humic Nitisols and Eutric Vertisols. The nutrients in the soils have been depleted. The farmers harvest the crops with the entire stalks leaving only the roots in the soils that gradually diminish the productivity of the land, and the buildup of soil acidity. The farmers usually request the office of agriculture to undertake evaluation of soil health status for management. The experimental site was therefore accessed from the farm land owned by the farmer whose productivity for crops showed declining from time to time.

### 2.2. Soil sampling, and analysis

Composite soil samples (0–20 cm) were collected in a rectangular design where four soil samples were taken at the corners while one at the center. These were repeated until all the experimental blocks are covered. Finally, 30 soil samples were collected and mixed to produce one composite sample for analysis of soil physical and chemical properties before planting. After harvest, however the samples were collected from each plot and replications comprising 36 composite soil samples following the same design. Samples were air-dried and then ground using a pestle and mortar to pass through a 2-mm sieve. Soil particle size distribution was determined using the Bouyoucos hydrometer method [22]. Bulk density was determined from the ratio of oven-dried soil to the bulk volume. Based on Van Reeuwijk’s [23] instruction, soil pH was measured from solution of 1: 2.5 soil to water ratio. Soil OC was determined using the Walkly-Black method [24], and a factor of 1.724 was applied to the result to determine the soil OM. Total N was measured using the micro-Kjeldahl method as outlined by Bremner and Mulvaney [25]. The Olsen method [26] was used to determine available P. Following 1 M ammonium acetate leaching, ethanol washing, and replacement of adsorbed ammonium with Na^+^ was performed on the soil samples to calculate the soil CEC [27].

### 2.3. Experimental design and management

Randomized complete block design (RCBD) was employed for this study having three replications, and twelve treatments. The experiment was constructed (factorial combination of 4 levels of manure × 3 levels of lime). Four weeks prior to planting, manure and lime were applied in a row and incorporated into the soil with the specified rate and mixed into the soil.

The field was leveled, divided into three blocks, each of which was then subdivided into twelve plots. Each plot had 3.75 m × 3.5 m gross dimension, and comprised 5 rows, each measuring 3 m in length. The distances between blocks and plots were 1 m and 0.5 m, respectively, and between rows and plants were 0.75 m and 0. 25 m. Random assignments of the treatments were made to each plot. On May 30, 2019, maize variety BH-661 seeds at a rate of 100 kg ha^-1^ were manually drilled. Every other agronomic practice, including disease, insect, and weed management, and hoeing were applied. Fertilizers such as NPSB blended which has a nutrient composition of (18.9% N, 37.7% P_2_O_5_, 6.95% S and 0.1% B) at 100 kg ha^-1^ was applied at planting while Urea at 100 kg ha^-1^ was applied in split in which half at planting and half dose at knee height stage of the crop. When the crop attained physiological maturity, the central rows were harvested. Then, it was sun-dried, and threshed to measure the grain yield.

### 2.4. Statistical analysis

Normality was checked before data analysis using the Shapiro-Wilk test. The F-test was employed to evaluate the homogeneity of error variance following the methodology described by Gomez and Gomez [28]. The analysis of variance was carried out using statistical analysis computer software SAS version 9.3 (Statistical Analysis System) [29]. The LSD test was employed at a 5% probability level to compare the means of all significant treatments when the ANOVA revealed significant difference (p < 0.05).

Partial budget analysis and marginal analysis were conducted following the CIMMYT methodology to evaluate the economic feasibility of the treatments, utilizing prevailing local market prices where grain yield (maize) was valued at 80.00 ETB per kg and stalk yield at 0.50 ETB per kg, alongside agricultural lime estimated at 2.00 ETB per kg and farmyard manure at 1.50 ETB per kg, with a 10% downward adjustment factor applied to experimental plot yields. Gross Field Benefit (GFB) was calculated by multiplying the field-adjusted grain and stalk yields by their respective unit prices per kg, while Total Variable Cost (TVC) summed the total expenses incurred for variable lime and manure inputs based on their per kg unit costs. Net Benefit (NB) was subsequently computed by subtracting the total variable cost from the gross field benefit for each treatment. Through dominance analysis, treatments were ranked in order of ascending total variable cost, and any treatment with a net benefit less than or equal to a preceding treatment with a lower or equal variable cost was designated as dominated and omitted from further calculations. Finally, for non-dominated treatments, the Marginal Rate of Return (MRR) was calculated as the percentage ratio of the change in net benefit to the change in total variable cost between consecutive treatments.

### 2.5. Ethics, consent to participate, and consent to publish declarations

This study did not involve human participants, human data, human tissues, animals, or any procedures requiring ethical approval. Therefore, ethics approval, consent to participate, and consent to publish are not applicable.

## 3. Results and discussion

### 3.1. Soil physicochemical properties and manure characterization of the experimental site

Based on the specified particle size proportions, clay was found as the structural class of the soil used for the experiment. The soil had a bulk density (BD) of 1.40 g cm^-3^ (Table 1). The bulk density of the soil was suitable for the growth of maize crops because it allowed for adequate aeration and water movement, and was less likely to hinder root development. The pH of the soil was 5.12, classified as strongly acid soil (Table 1). The observed low values of exchangeable acidity in such strongly acid soils could be the medium CEC found in the soil, the strong attachment of exchangeable Al^+^ and H^+^ on the crystal lattice, or the type of clay mineralogy and parent materials [30]. According to Baquy et al. [31], the critical pH and exchangeable Al ranges from 4.46–5.07, and, 1.99, 1.93, and 1.04–2.74 cmol_c_ kg^-1^ respectively, for maize production.

**Table 1:** Physicochemical properties of the experimental field soil before sowing.

Soil properties	Values	Rating	Reference
Sand (%)	20		
Silt (%)	33		
Clay (%)	47		
Textural class	Clay		
BD g cm^-3^	1.42	Favorable	Hazelton and Murphy, 2016
pH (H_2_O) Exchangeable acidity (Cmole_c_ kg^-1^)	5.12 1.4	Strongly acid	Hazelton and Murphy, 2016
Total N %	0.11	Low	Landon, 2014
Available P mg kg^-1^	8.81	Very low	Olsen et al, 1954
Organic carbon (%)	1.21	Very low	Landon, 2014
Organic matter (%)	2.27	Very low	
CEC (cmol_c_ kg^-1^)	19.27	Medium	Landon, 2014
Exchangeable acidity (cmol_c_ kg^-1^)	0.95	Medium	Karltun et al, 2013

Such an acidic level hinders maize growth and productivity due to toxicity of Al, deficiencies of basic cations, and reduced availability of P [32]. Thus, it was established that acid soil management strategies were necessary for the experimental soil. According to Landon [33] rating of soil test values, the soil OC, and total N contents were very low, and low, respectively. This may be the result of all the plant biomass and root residue being removed from the soil. According to Musinguzi et al. [34], tropical soils must have OC content of 1.9–2.2% in order to be considered productive and to have favorable structural condition and stability. In the study area’s maize-growing fields, where there is no residue in the soil, lower fertilizer application rates, and continuous cultivation that promotes a higher rate of mineralization, declined soil OC and total N contents are anticipated [35].

In the clay-textured soils of the research area, available P was extremely low (8.81 mg kg^-1^), which is less than the critical value (30 mg kg^-1^) at which maize has accessible P as recommended by EthioSIS [36]. This could be ascribed to the soil’s acidity, low rates of P-containing fertilizer application, crop uptake that occurs continuously, and erosion in the highland. Based on Landon [33] ratings, the soils in the study have CEC value of 19.27 cmolc kg^-1^, indicating a moderate fertility status of the soil. The manure was analyzed and the results were indicated in Table 2.

**Table 2:** Some chemical contents of FYM used for the experiment.

Nutrient source	N (%)	P (%)	K (%)	OC	OM	pH
Manure	0.76	1.12	1.81	21.4	36.2	6.8

### 3.2. Effects of manure and lime on tasseling, silking and physiological maturity

The application of manure or lime significantly (P ≤ 0.01) affected the number of days to 50% tasseling, silking, and physiological maturity (Table 3). Longer duration was taken for all these phenological variables when the plots received highest levels of manure. In contrast, the control plot took shorter number of days for 50% tasseling (Table 3). The longer duration of tasseling, silking, and physiological maturity at higher rate of manure could be related to increased nutrient availability, which postponed the growth stage of tasseling and silking. The days of tasseling were greatly impacted by the amounts of manure [37] which may be due to the slow release of nutrients throughout the growing period that could not give quick tasseling and silking. In a similar report, Yohannes [38] reported that a higher quantity of manure increased crop biomass because it made more nutrients available, lengthening the crop’s growing season and adding days until silking.

**Table 3:** Main effects of lime and manure levels on DTT, DTS, DTM.

Treatments	DTT	DTS	DTM
**Manure levels (t ha**^**-1**^)			
**0**	72.8c	76.9d	143.6d
**4**	75.0c	79.4c	145.9c
**8**	78.0b	83.5b	148.8b
**12**	84.1a	89.8a	153.3a
**F-test**	29.73	52.55	57.62
**P-value**	0.0001	0.0001	0.0001
**LSD**	2.69	2.27	1.62
**Lime levels (t ha**^**-1**^)			
**0**	75.2b	79.6c	146.9
**1.5**	77.3b	85.2a	147.3
**3**	79.9a	82.4b	149.4
**F-test**	9.28	16.75	7.77
**P-value**	0.001	0.0001	0.002
**LSD**	2.64	1.97	1.46
**CV (%)**	2.05	1.63	0.65

Means within a column followed by the same letter are not significantly different from each other at P ≤ 0.05. DTT = Days to tasseling; DTS = Days to silking; DTM = Days to physiological maturity; LSD = Least significant difference; ns = Nonsignificant difference.

More number of days (153.3) were taken to attain 90% physiological maturity from the application of 12 t ha^-1^ manure, while shortest days (143.6) to the control plots (Table 3). The days to physiological maturity increased numerically along with the manure rates. Lime application alone did not show significant variation to maturity dates.

Rahman et al. [39] claimed that blending compost with mineral fertilizer produced the highest level of maturity. Bekele et al [40] reported physiological maturity of maize was considerably prolonged when lime was applied together with vermicompost and inorganic P fertilizer.

### 3.3. Effects of manure and lime on growth, and yield parameters

#### 3.3.1. Plant height and number of leaves per plant.

Application of manure had a highly significant (p ≤ 0.01) effect on maize plant height (Table 4). However, lime alone or its interaction with manure was found nonsignificant to plant height plant height, and leaves per plant. The tallest mean plant height (226.8 cm) was obtained from plots received 12 t manure ha^-1^ while the shortest mean plant height (206.2 cm) was observed from the control plots. An increase in manure fertilizer rates caused a significant increase in the mean plant height of maize. This resulted from both the plant’s ability to absorb N from manure and the increased OM in the soil. According to Mhoro [20], increasing N rates resulted in a significant variance in plant height at harvest. Likewise notable rise in the height of maize plants was observed when the rates of manure application increased [11,41]. This could be due to the prolonged vegetative growth period of maize, and boosts the production of photosynthetic assimilate and its partitioning to stems, which may have positive effects on the height of the crop.

**Table 4:** Main effects of lime and manure levels on DTT, DTS, DTM.

Treatments	PH (cm)	NLPP	NCPP	NGPC	TGW
**Manure levels (t ha**^**-1**^)					
**0**	206.2	8.1	1.4	281.2	310.1
**4**	213.7	9.4	2.1	331.1	321.9
**8**	219.8	10.4	2.4	379.8	345.2
**12**	226.8	11.9	3.3	404.9	618.4
**F-test**	182.27	47.18	123.32	81.63	0.96
**P-value**	0.0001	0.0001	0.0001	0.0001	0.43
**LSD**	1.90	0.69	0.21	17.76	10.92
**Lime levels (t ha**^**-1**^)					
**0**	216.1	9.5	2.1	323.0	314.8
**1.5**	217.1	10.6	2.2	350.8	327.2
**3**	216.4	9.6	2.6	373.9	554.8
**F-test**	0.76	8.73	20.61	23.62	1.08
**P-value**	0.48	0.002	0.0001	0.0001	0.36
**LSD**	1.64	0.59	0.18	15.39	38.16
**CV (%)**	0.52	4.1	5.39	3.00	1.62

Means within a column followed by the same letter are not significantly different from each other at P ≤ 0.05. PH = Plant height; NLPP = Number of leaves per plant; NCPP = Number of cobs per plant; TGW = Thousand grain weight; LSD = Least significant difference, CV: coefficient of variation.

Application of manure had a significant (p ≤ 0.01) improvement on the number of leaves per plant (NLPP). Plots treated with 12 t ha^-1^ had the maximum mean number of leaves (11.9), and the low mean number of leaves per plant (8.1) from the control group (Table 4). The observed more number of leaves from manure application may be related with the supply of N nutrition to the plant. Thus, the positive effect of N on vigorous vegetative growth and inter-nodal extension may account for the increase in leaf count with increasing the rates of manure application. The number of leaves per plant affects its photosynthetic activity that in turn affects crop growth and yield. Improved canopy and weed suppression were also reported from the increased number of leaves from each plant [42].

#### 3.3.2. Number of cobs per plant, grain per cob, and thousand grain weights.

The main and interaction effects of manure with lime significantly (p ≤ 0.01) affected the number of cobs per plant and grains per cob (Table 4 and Table 5). However, thousand grain weights were not significantly affected by the main or the interaction effects of the treatments. Highest mean number of cobs (3.3) was counted from the addition of 12 t manure ha^-1^, while the least (1.4) was from the control group. Lime and manure might contribute to improved soil condition which may be the cause for increased grains per cob production. Singh and Misal [43] explained that addition of FYM and vermicompost significantly increased cob number and grain per cob. They attributed the provision of N from the decomposition organic fertilizers to cause the improvement. Plots treated with 3 t lime ha^-1^ produced significant number of grains in each cob (2.327). The possible explanation for this could be the role that calcium, which is present in lime, plays in cell growth and division, membrane permeability, enzyme activation, and cell protection from external toxins.

**Table 5:** Interaction effects of lime and manure on cobs per plant and grain per cop.

Lime levels (t ha^-1^)	Manure levels (t ha^-1^)			
	0	4	8	12
**CPP**				
**0**	1.1g	1.8f	2.3de	3.1ab
**1.5**	1.0g	2.0ef	2.2def	3.5a
**3**	2.0ef	2.5dc	2.8bc	3.1ab
**F-test**	5.73			
**P-value**	0.001			
**LSD**	0.4			
**CV (%)**	10.35)			
**GPC**				
**0**	225.0h	300.0fg	373.3bcd	393.7abc
**1.5**	298.0g	341.6def	366.7bcd	397.0ab
**3**	320.6efg	351.7cde	399.3ab	424.0a
**F-test**	3.71			
**P-value**	0.01			
**LSD**	42.9			
**CV (%)**	7.25			

Means within a column followed by the same letter are not significantly different from each other at P ≤ 0.05. CPP = Cobs per plant; GPC = Grain per cob; LSD = Least significant difference: CV: coefficient of variation.

The number of cobs per plant and grains per cobs were significantly affected by the interaction between the lime rate and the manure fertilizer (Table 5). Plots treated with 12 t ha^-1^ manure combined with 1.5 t lime ha^-1^demonstated higher values of NCPP and NGPC compared to the control. The observed significant rise in NCPP and NGPC with increased manure and lime rate could potentially be attributed to the synergistic effects of the amendment, which reduce soil acidity, enhance nutrient use efficiencies and promote stem and cob development in maize. The reduced mobility of aluminum, the increased pH, availability of P and basic cations reflected in the improvement maize growth characteristics. Cattle manure could also reduce acidity due to its pronounced buffering capacity and content of adsorbed base cations [44]. Wakwoya et al. [19] also indicated increase in soil pH due to lime with a reduced solubility of plant toxic nutrients like Fe^+3^, Al^+3^, Mn^+2^ and B.

#### 3.3.3. Grain yield, stalk yield, and aboveground dry biomass.

Grain yield, stalk yield, and aboveground biomass yields were affected significantly (P < 0.01) due to application of manure, lime, and their interactions (Fig 1). The greatest grain yield (4.41 t ha^-1^) was obtained from the combinations of 3 t lime ha^-1^ with 8 t manure ha^-1^ followed by 3 t lime ha^-1^ with 12 t manure ha^-1^. The lowest grain yield (1.02 t ha^-1^) was obtained from the control. The grain yield advantage obtained from the above treatment combinations were 332.4%, and 233.3%, respectively over the control. In the same way, highest stalk and dry aboveground biomass yields were achieved from 3 t lime ha^-1^ with 8 t manure application. Due to increased number of cobs per plant and grains per cob, the grain yield of individual maize plant was improved with integration of manure, and lime. Likewise, significant yield increase of 210% and 181% in maize was reported for Cambisols and Nitisols, respectively at Kellem Wollega Zone of western Ethiopia [45]. Another experiment conducted in Kembata Zone, highest agronomic yield was achieved with 5.8 t ha^−1^ lime and 15 t ha^−1^ FYM, while the economic optimum for resource poor farmers was recommended at 5.8 t ha^−1^ lime and 10 t ha^−1^ FYM [46].

**Fig 1:**
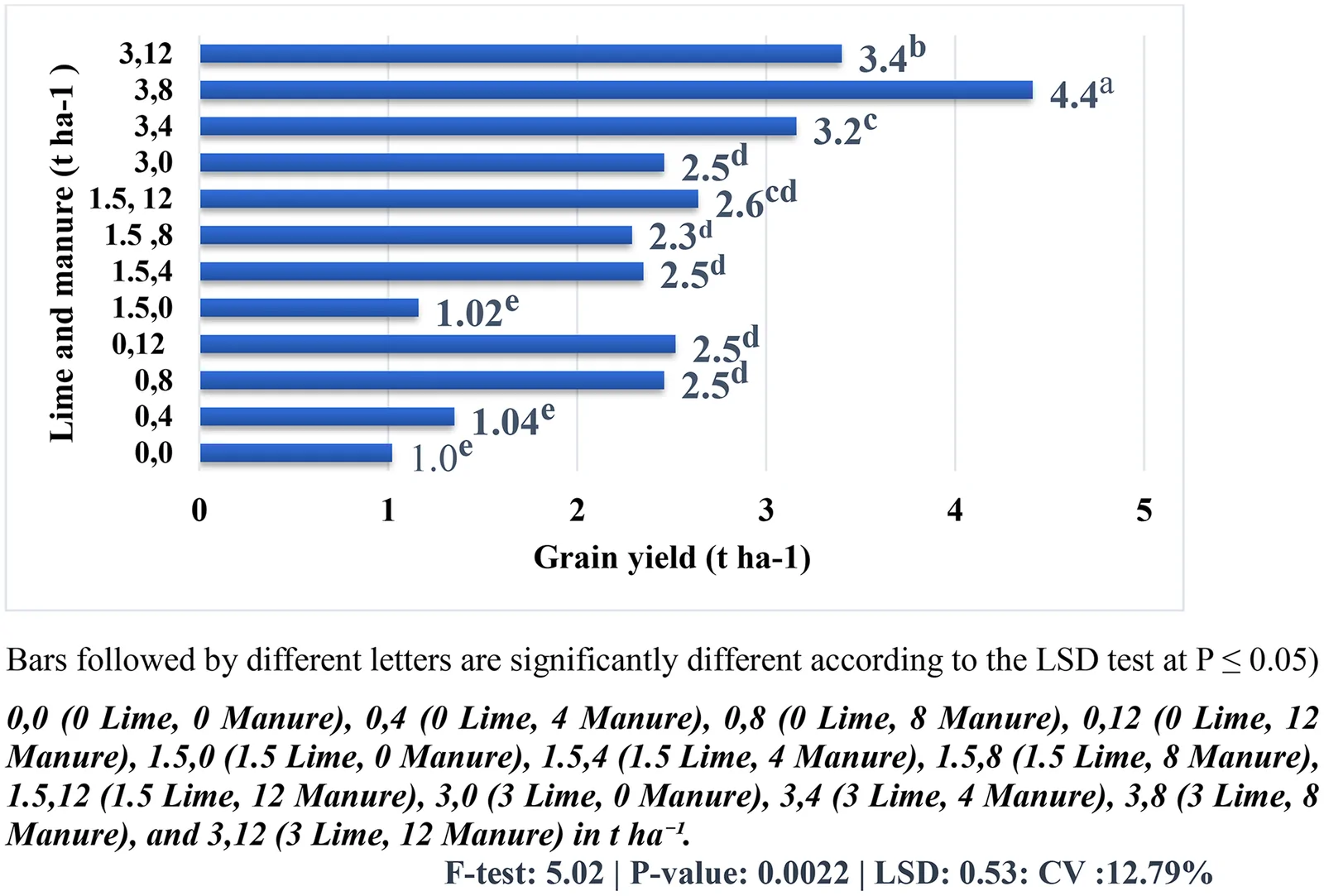
Interaction effect of lime and manure rates on maize grain yield.

Rajesh et al [47] reported that the variations in the grain yield and yield components could be related to the growth components of maize such as plant height, number of leaves per plant, leaf area index.

The observed significant yield in this experiment could be the associated to the response of maize to the added manure in the soil which was poor in its native N and P contents. The application of manure also played the principal role in improving soil structure and moisture conservation. The improved soil pH due to application of lime also made nutrients like P more readily available and enhance the productivity of maize [32]. This finding was consistent with Kimiti et al [48] who found that the application of lime, manure combined with mineral fertilizers increased maize crop yield when compared to the application of mineral fertilizers, lime, and manure alone. In the same way, the application of 2.5 t ha^-1^ lime on acid Luvisols reduced the aluminum content below the toxicity limit, while more aluminum was found in the control treatment. The observed positive change in soil chemical properties (OC, total N, and available P) after the crop harvest justified the yield increase.

Aboveground dry biomass and stalk yield were linearly improved with increasing manure rates (Figs 2 and 3). The released N from manure contributed to the increase in height, leaves, and cobs of maize, which eventually improved the total biomass of the plant. Furthermore, the ameliorating effects of lime on soil acidity and positive effect of lime application on the overall soil properties might cause improved biomass [49]. The increased yield of AGDB may be the result of liming and organic fertilizers releasing unavailable nutrients from highly acidic soil. Plants may have access to nutrients in the soil when acidity is reduced. This significant variation in total AGDB might be also associated with the result of the beneficial interactions between lime and manure fertilizer, as well as high doses of both substances, which are known to promote soil CEC, OM, SOC, and plant vegetative growth. The mineralization of manure released Ca^2+^ that displaced Al^3+^ which in turn reacts with water to form insoluble Al(OH)_3_ and water. Other researchers [14,50,51] also reported the effectiveness of integrated application of lime and manure on different crops.

**Fig 2:**
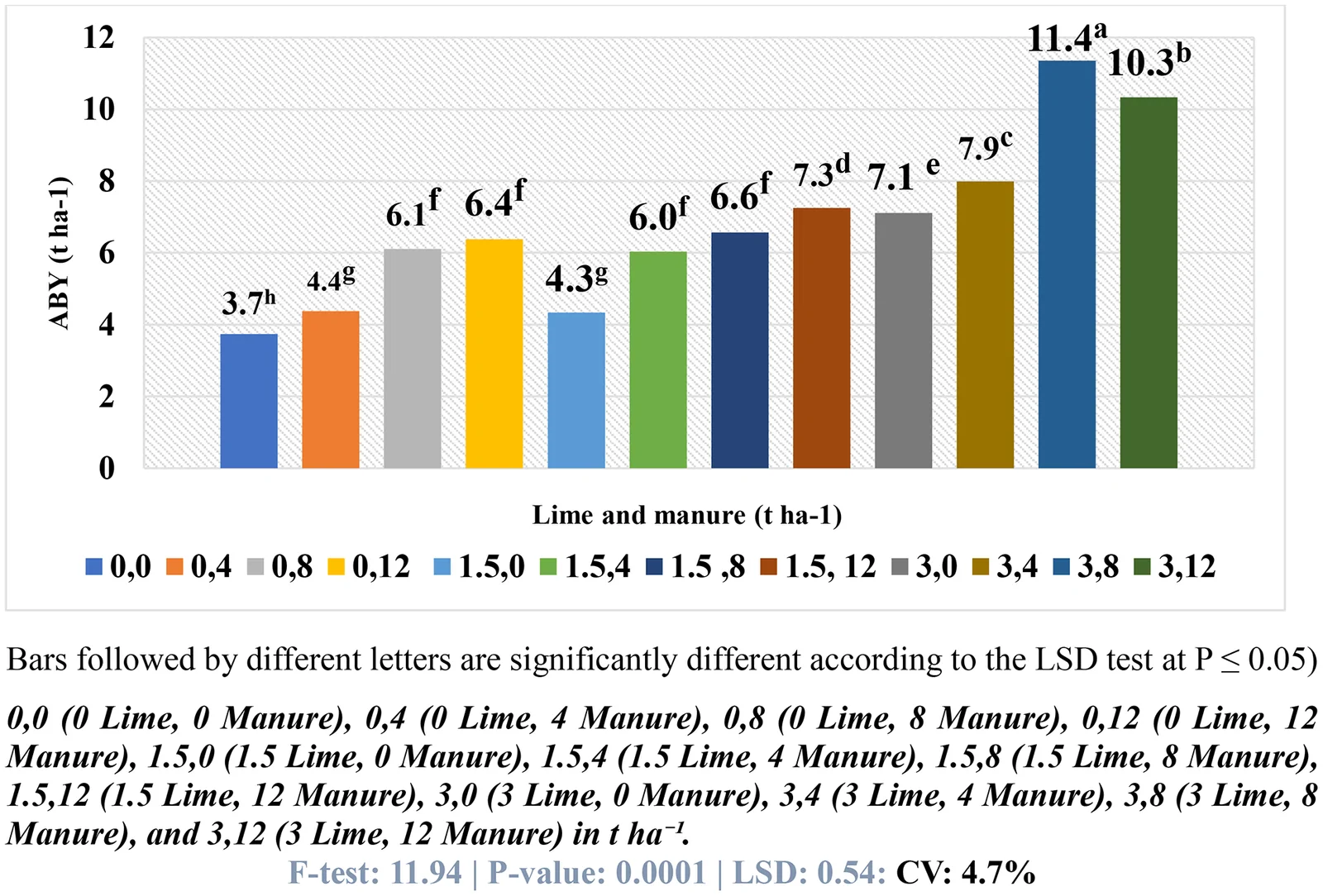
Aboveground biomass (ABY) as influenced by the interaction effects of lime and manure.

**Fig 3:**
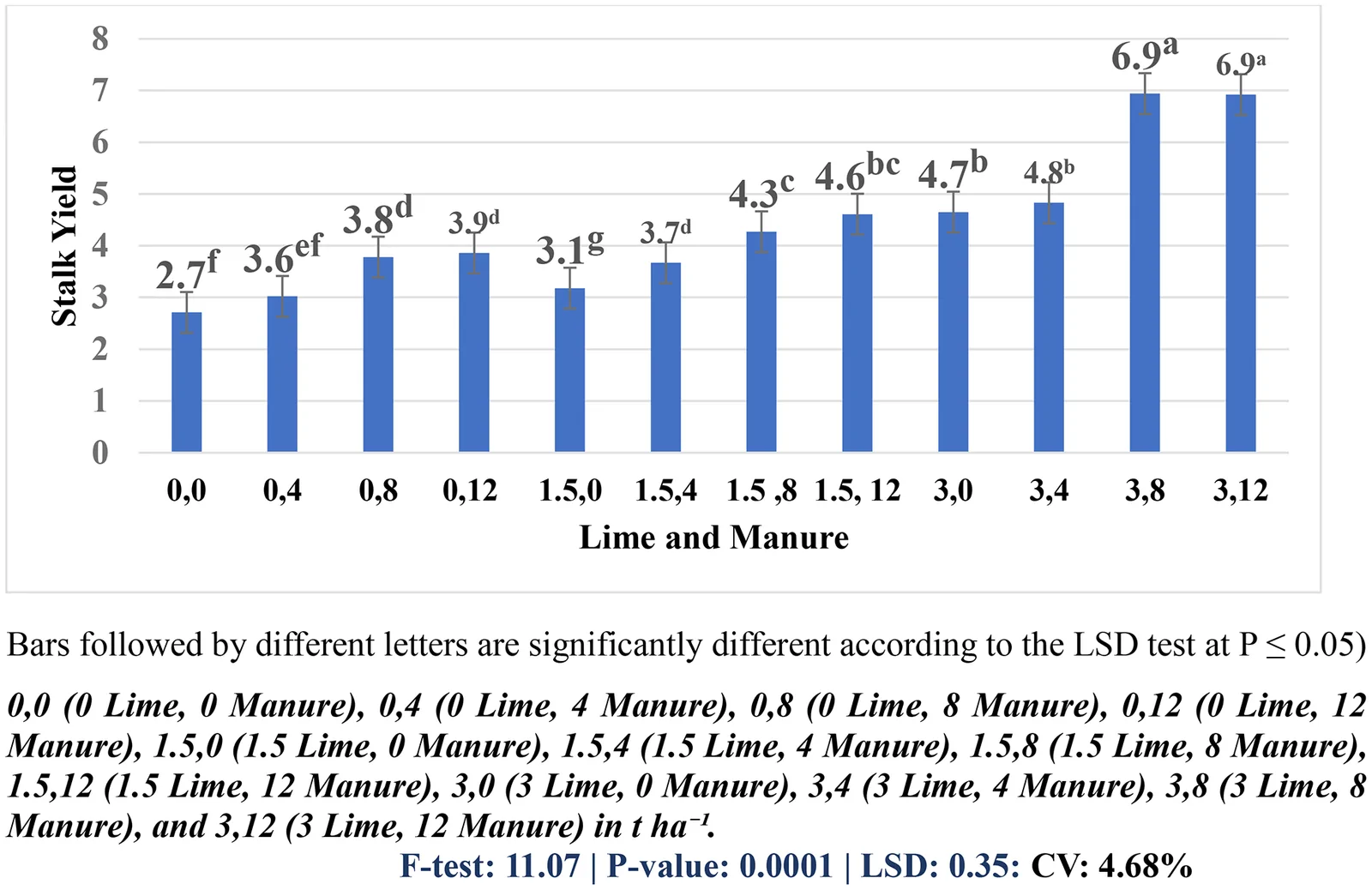
Interaction effects of lime and manure on stalk yield.

### 3.4. Change in soil pH, OC, TN and Available P after harvest

In comparison to the control, this experiment showed that integrated nutrient management had an impact on pH, which was raised with the application of lime and manure fertilizer (Table 6). Plots treated separately with 12 t ha^-1^ manure, and 3 t ha^-1^ lime showed relatively higher pH values as compared to their respective controls. Combine application of 12 t ha^-1^manure and 3 t ha^-1^ lime raised soil pH by 0.8 units over the control (Fig 4).

**Table 6:** Main effects of lime and manure levels on soil pH, SOC, TN, AVP, CEC, and Ex Acidity.

Treatments Manure levels (t ha^-1^)	pH (cm)	SOC	TN	AVP	CEC	Ex Ac
**0**	5.30d	1.27d	0.11a	9.15d	23.38c	1.56a
**4**	5.39c	1.51c	0.18a	10.10c	25.78b	1.39b
**8**	5.42b	1.77b	0.22a	10.79b	28.56a	1.35b
**12**	5.53a	1.96a	0.26a	11.85a	29.17a	1.19c
**F-test**	23.79	148.84	1.19	89.52	97.24	27.63
**P-value**	0.0001	0.0001	0.34	0.0001	0.0001	0.0001
**LSD**	0.05	0.07	0.16	0.35	0.79	0.08
**Lime levels (t ha**^**-1**^)						
**0**	5.11a	1.46a	0.24	8.6c	26.22	1.75a
**1.5**	5.47b	1.66b	0.17	10.57b	26.69	1.44b
**3**	5.64c	1.75c	0.16	12.20a	27.26	0.95c
**F-test**	248.54	**	0.88	297.56	4.85	265.43
**P-value**	0.0001	48.45	0.43	0.0001	0.02	0.0001
**LSD**	0.05	0.06	0.14	0.30	0.68	0.07
**CV (%)**	0.55	2.54	49.9	1.97	1.75	3.4

Means within a column followed by the same letter are not significantly different from each other at P ≤ 0.05. SOC = Soil organic carbon; TN = Total nitrogen; AVP = Available phosphorus; CEC = Cation exchange capacity; Ex Ac = Exchangeable acidity; LSD = Least significant difference; ns = Nonsignificant difference.

**Fig 4:**
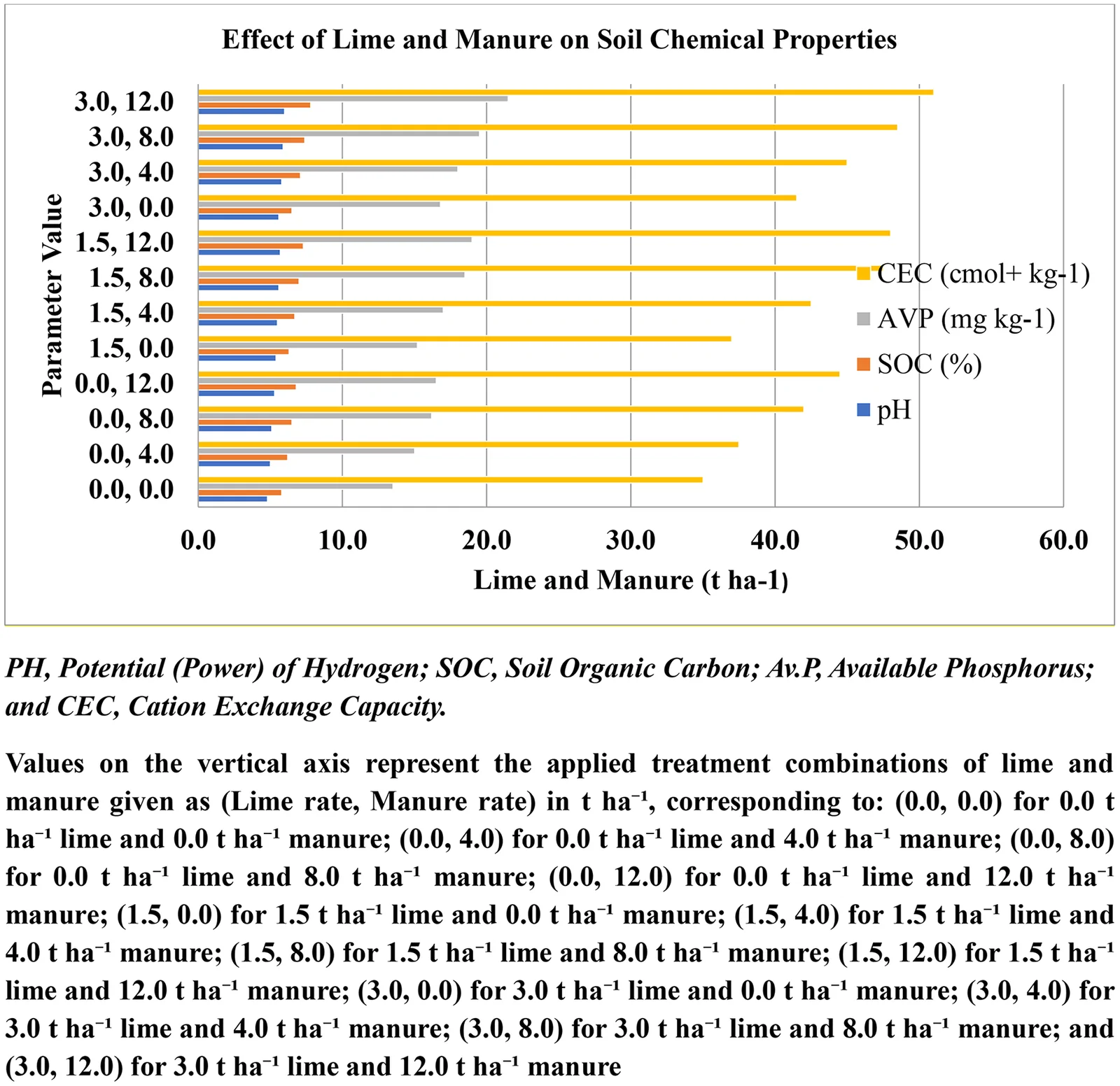
Interaction effects of lime and manure on soil chemical properties after harvesting.

Meena et al. [8] noted continuous application of FYM in acidic soil brought an increase in pH. In the same way, most research findings indicated that manure addition increased soil pH [10,49], especially when the initial pH is acid. They justified that most manure produces basic ions, and also forms complexation with acid ions like Al. However, the type and amount of manure, degree of incorporation, and initial soil properties likely determine the influence of manure on soil pH [52].

The pH of the soil was successfully raised from 5.11 to 5.64 by applying lime alone. Because lime contains basic cations (Ca^2+^) and anions (CO_3_^2-^), which can exchange H^+^ ions from exchangeable sites to form (H_2_O + CO_3_^2-^). Applying lime increases the pH of the soil, which creates an environment that is more conducive to soil microbiological movement and reproduction, speeds up the release of plant nutrients. Liming reduced acidity and enhanced nutrient availability, primarily phosphorus [10,13]. Post-harvest soil analysis showed that the highest soil pH value (5.67) was measured from 3.72 t lime ha^-1^ in western Ethiopia.

The plots supplied with 12 t manure ha^-1^ only had the highest OC content (1.96%), which was an increase of 54% from the initial soil OC. When plant biomass is added, the breakdown of dead plant material, such as roots and leaves, may be linked to an increase in SOC. Comparable results were reported to show increased soil OC after the application of lime integrated with inorganic and organic fertilizers on maize plots [53]. Similar result was obtained by Moges et al. [54] who observed that an increase in OC from 2.23 to 2.49% following the application of lime with inorganic fertilizers in the Angolela Tera district. On the other hand, SOC decreased from 1.32 to 1.29% with the application of lime only.

Lime or manure application did not show significant variation in soil total N after the crop harvest (Table 6). However, based on the status rating criteria suggested by Tekalign [55], the total N content in the soils treated with 12 t ha^-1^ manure shifted the low status total N to the high status. Because of this, it is evident that applying organic fertilizers raised total N, which could be related to the mineralization of N from OM decomposition. Feedlot manure was effective in correcting deficient or low N, P, or Mg status [56]. There have also been reports from various authors in Ethiopia of an increase in total N due to application of integrated fertilizers [57].

Following 3 t ha^-1^ lime with 12 t ha^-1^ manure applications, the available P content was found to be significantly the highest (14.41 mg kg^-1^). Because clay soil naturally contains P reserves that have been mineralized or dissolved, the application of lime and organic fertilizers together in this acid soils has increased the amount of P that is available in the soil. Thus, increasing crop P utilization from leftover fertilizer is one of the main advantages of liming acid soils. Several researchers indicated the increase in available P in soils received lime and manure [58–60]. This might be due to the release of Ca^2+^and OH^-^ from hydrolysis reaction in soil moisture. The generated Ca^2+^exchanges Al^3+^ while the OH^-^ reacts with Al^3+^ and H^+^ to produce water and aluminum hydroxide, respectively. The pH of the soil was raised by the OH^-^, which lowered the exchangeable acidity [61,62]. However, manure chelating substances that could complex with exchangeable Al to reduce its toxicity, and P fixation.

### 3.5. Change in soil CEC and exchangeable acidity after harvest

Cation exchange capacity was influenced significantly with the application of manure as compared to the control. The number of negative charges on the clay or organic matter particles may have increased due to an increased pH, which would also increase CEC. In agreement with this finding, many researchers showed increase in CEC of soils due to organic matter application [63,64]. Separate application of 12 t manure ha^-1^ or 3 t lime ha^-1^ significantly reduced the exchangeable acidity of the soil. The reasons for the observed variations have been discussed in the soil pH section above.

### 3.6. Partial budget and dominance analysis of maize under Different Lime and Manure applications

The partial budget, dominance, and marginal rate of return (MRR) analyses demonstrated substantial economic differences among the evaluated lime and manure combinations (Tables 7 and 8). The untreated control (0 t ha^−1^ lime + 0 t ha^−1^ manure) produced the lowest net benefit (ETB 74,659.50), providing the baseline for economic comparison (Table 7). Progressive application of lime and manure generally increased gross field benefits and net returns, although not all treatment combinations were economically efficient.

**Table 7:** Partial budget and dominance analysis of maize under different lime and manure applications.

Lime (t ha − 1)	Manure (t ha − 1)	Adj. Grain Yield (kg ha − 1)	Adj. Stalk Yield (kg ha − 1)	Gross Field Benefit (ETB)	Total Variable Cost (ETB)	Net Benefit (ETB)	
0	**0**	918.0	2,439.0	74,659.50	0.00	74,659.50	
1.5	**0**	1,044.0	2,862.0	84,951.00	3,000.00	81,951.00	
0	**4**	1,215.0	2,718.0	98,559.00	6,000.00	92,559.00	
3	**0**	2,214.0	4,185.0	179,212.50	6,000.00	173,212.50	
1.5	**4**	2,115.0	3,303.0	170,851.50	9,000.00	161,851.50	D
0	**8**	2,097.0	3,402.0	169,461.00	12,000.00	157,461.00	D
3	**4**	2,844.0	4,347.0	229,693.50	12,000.00	217,693.50	D
1.5	**8**	2,061.0	3,843.0	166,801.50	15,000.00	151,801.50	D
0	**12**	2,268.0	3,474.0	183,177.00	18,000.00	165,177.00	
3	**8**	3,969.0	6,246.0	320,643.00	18,000.00	302,643.00	
1.5	**12**	2,376.0	4,149.0	192,154.50	21,000.00	171,154.50	D
3	**12**	3,060.0	6,228.0	247,914.00	24,000.00	223,914.00	

**Table 8:** Marginal rate of return (MRR) analysis.

Treatment (Lime + Manure)	Total Variable Cost (ETB)	Net Benefit (ETB)	Change in Net Benefit (ETB)	Change in TVC (ETB)	Marginal Rate of Return (MRR %)
Lime 0, Manure 0	0.00	74,659.50	—	—	—
Lime 1.5, Manure 0	3,000.00	81,951.00	7,291.50	3,000.00	**243.05%**
Lime 3, Manure 0	6,000.00	173,212.50	91,261.50	3,000.00	**3,042.05%**
Lime 3, Manure 4	12,000.00	217,693.50	44,481.00	6,000.00	**741.35%**
Lime 3, Manure 8	18,000.00	302,643.00	84,949.50	6,000.00	**1,415.83%**

Dominance analysis identified four treatments (1.5 t ha^−1^ lime + 4 t ha^−1^ manure, 0 t ha^−1^ lime + 8 t ha^−1^ manure, 3 t ha^−1^ lime + 4 t ha^−1^ manure, and 1.5 t ha^−1^ lime + 12 t ha^−1^ manure) as economically dominated because they incurred higher production costs without generating proportional increases in net benefits relative to less costly alternatives (Table 7). According to the CIMMYT partial budget procedure, such treatments are excluded from further economic consideration because they are not rational investment options for farmers.

After eliminating dominated treatments, the remaining non-dominated treatments consistently produced acceptable economic returns (Table 8). Applying 1.5 t ha^−1^ lime alone increased the net benefit by ETB 7,291.50 over the control and achieved an MRR of 243.05%, indicating that each additional ETB invested returned ETB 2.43 above the initial investment. Increasing the lime rate to 3 t ha^−1^ without manure substantially improved profitability, generating a net benefit of ETB 173,212.50 and the highest MRR (3,042.05%), reflecting exceptional economic efficiency.

Further addition of manure to 3 t ha^−1^ lime continued to increase economic returns despite the higher production costs. The combination of 3 t ha^−1^ lime with 4 t ha^−1^ manure produced a net benefit of ETB 217,693.50 with an MRR of 741.35%, while increasing manure to 8 t ha^−1^ resulted in the highest net benefit (ETB 302,643.00) and an MRR of 1,415.83% (Table 8). These MRR values greatly exceeded the minimum acceptable rate of return (100%) commonly recommended for smallholder farming systems, indicating that the additional investment was economically justified.

## 4. Conclusions

The applications of various rates of lime and manure showed different results in maize growth, and yield, as well as post-harvest soil properties. The highest rate manure application was effective in improving the growth of plant height, and leaves. Grain and total biomass yields of maize were significantly increased when 3 t lime ha^-1^ was applied with 8 manure t ha^-1^. Manure application increased the soil carbon content after harvest. Soil pH, available P, and CEC were improved while exchangeable acidity was reduced. The observed increase in maize yield could be due to the positive influence of lime and manure in reducing soil acidity while improving the availability of soil nutrients. Similar works over locations and years with economic analysis should be done for valid recommendation of the amendments on acid soils and enhanced maize productivity. the yield economic analysis revealed that integrating 3 t ha^−1^ lime with 8 t ha^−1^ manure provided the greatest yield and financial return among the evaluated treatments, whereas application of 3 t ha^−1^ lime alone offered the highest investment efficiency. Therefore, under the prevailing production conditions, the combined application of 3 t ha^−1^ lime and 8 t ha^−1^ manure can be recommended as the most profitable management option because it maximized net economic benefits while maintaining an economically attractive marginal rate of return

## Supporting information

S1 File Maize growth data. (XLSX)

S2 File Soil data. (DOCX)

S3 File Yield data. (DOCX)

## References

[1] Bello WB. Influence of gypsum application on wheat (Triticum aestivum L.) yield and components on saline and alkaline soils of Tigray Region, Ethiopia. Green J Agri Sci. 2012;2(7):311–7.

[2] FAO. FAOSTAT: Statistical Database. Rome, Italy: Food and Agriculture Organization of the United Nations; 2021. Available from: https://www.fao.org/faostat/

[3] Gachene CKK, Karuma AN, Baaru MW. Climate change and crop yield in Sub-Saharan Africa. In: Lal R, Singh B, Mwaseba D, Kraybill D, Hansen D, Eik L, editors. Sustainable Intensification to Advance Food Security and Enhance Climate Resilience in Africa. Cham: Springer; 2015. pp. 129–45. doi: 10.1007/978-3-319-09360-4_8

[4] Regassa A, Assen M, Ali A, Gessesse B. Major Soil Types. In: Beyene S, Regassa A, Mishra BB, Haile M, editors. World Soils Book Series. Cham: Springer International Publishing. 2023. pp. 77–110. doi: 10.1007/978-3-031-17012-6_6

[5] Agegnehu G, Amede T, Erkossa T, Yirga C, Henry C, Tyler R, et al. Extent and management of acid soils for sustainable crop production system in the tropical agroecosystems: a review. Acta Agric Scand Section B — Soil Plant Sci. 2021;71(9):852–69. doi: 10.1080/09064710.2021.1954239

[6] Kunito T, Isomura I, Sumi H, Park H-D, Toda H, Otsuka S, et al. Aluminum and acidity suppress microbial activity and biomass in acidic forest soils. Soil Biol Biochem. 2016;97:23–30. doi: 10.1016/j.soilbio.2016.02.019

[7] Baquy MA-A, Li J, Nkoh JN, Biswash MdR, Xu R-K. Determining critical soil pH for phosphorus uptake efficiency in an acidic Ultisol for maize. Egypt J Soil Sci. 2024;64(4):1525–36. doi: 10.21608/ejss.2024.308115.1827

[8] Meena HM, Sharma RP, Sankhyan NK, Sepehya S. Effect of continuous application of fertilizers, farmyard manure and lime on soil fertility and productivity of the maize-wheat system in an acid alfisol. Commun Soil Sci Plant Anal. 2017;48(13):1552–63. doi: 10.1080/00103624.2017.1373800

[9] Chauhan N, Sankhyan NK, Sharma RP, Singh J, Gourav. Effect of long-term application of inorganic fertilizers, farm yard manure and lime on wheat (*Triticum aestivum* L.) productivity, quality and nutrient content in an acid alfisol. J Plant Nutr. 2020;43(17):2569–78. doi: 10.1080/01904167.2020.1783298

[10] Fekadu E, Kibret K, Bedadi B, Melese A, Yitaferu B. Organic and inorganic amendments on soil chemical properties at different period of incubation of acidic soil. Euras J Soil Sci (EJSS). 2018;7(3):273–83. doi: 10.18393/ejss.435095

[11] Masood S, Naz T, Javed MT, Ahmed I, Ullah H, Iqbal M. Effect of short-term supply of farmyard manure on maize growth and soil parameters in pot culture. Arch Agron Soil Sci. 2013;60(3):337–47. doi: 10.1080/03650340.2013.792990

[12] Wenyika P, Enesi RO, Gorim LY, Dyck M. Effects of liming on soil physical and chemical properties in Europe and North America: A review. Agrosyst Geosci Environ. 2025;8(3). doi: 10.1002/agg2.70175

[13] Dinkecha K. Effects of liming on physicochemical properties and nutrient availability of acidic soils in Welmera Woreda, Central Highlands of Ethiopia. Biochem Mol Biol. 2017;2(6):102. doi: 10.11648/j.bmb.20170206.16

[14] Demissie W, Kidanu S, Abera T. Effects of lime, blended fertilizer (NPSB) and compost on yield and yield attributes of barley (Hordeum vulgare L.) on acid soils of Wolmera District, West Shoa, Ethiopia. Ethiop J Appl Sci Technol. 2017;8(2):84–100.

[15] Abate T, Shiferaw B, Menkir A, Wegary D, Kebede Y, Tesfaye K, et al. Factors that transformed maize productivity in Ethiopia. Food Sec. 2015;7(5):965–81. doi: 10.1007/s12571-015-0488-z

[16] Central Statistical Agency (CSA). Agricultural Sample Survey 2016/17: Report on Area and Production of Major Crops. Volume I. Addis Ababa, Ethiopia: CSA; 2017.

[17] Getachew A, Chilot B, Teklu E. Soil Acidity Management. Addis Ababa, Ethiopia: Ethiopian Institute of Agricultural Research (EIAR); 2019.

[18] Kumar M, Hazarika S, Choudhury BU, Ramesh T, Verma BC, Bordoloi LJ. Liming and integrated nutrient management for enhancing maize productivity on acidic soils of northeast India. Ind J Hill Farm. 2012;25(1):35–7.

[19] Wakwoya MB, Woldeyohannis WH, Yimamu FK. Effects of minimum tillage and liming on maize (Zea mays L.) yield components and selected properties of acid soils in Assosa Zone, West Ethiopia. J Agric Food Res. 2022;8:100301. doi: 10.1016/j.jafr.2022.100301

[20] Mhoro L, Meya AI, Amuri NA, Ndakidemi PA, Njau KN, Mtei KM. Potential of manure and urea fertilizer on maize (*Zea mays* L.) productivity and soil quality in the Northern Highlands of Tanzania. Agronomy. 2025;15(2):333. doi: 10.3390/agronomy15020333

[21] Asnake G, Mammo G. Farming climate change adaptation mechanisms and factors affecting farmers’ decision to adaptation: A case study in Meket District, North Wollo, Ethiopia. Int J Adv Sci Tech Res. 2016;6(2):87–114.

[22] Day PR. Hydrometer method of particle size analysis. In: Black CA, editor. Methods of Soil Analysis. Part 1: Physical and Mineralogical Properties, Including Statistics of Measurement and Sampling. Madison, WI: American Society of Agronomy; 1965. pp. 562–6.

[23] Van Reeuwijk LP. Procedures for Soil Analysis. 4th ed. Wageningen, The Netherlands: International Soil Reference and Information Centre (ISRIC); 1993.

[24] Walkley A, Black IA. An examination of the degtjareff method for determining soil organic matter, and a proposed modification of the chromic acid titration method. Soil Sci. 1934;37(1):29–38. doi: 10.1097/00010694-193401000-00003

[25] Bremner JM, Mulvaney CS. Nitrogen—Total. In: Page AL, Miller RH, Keeney DR, editors. Methods of Soil Analysis. Part 2: Chemical and Microbiological Properties. Madison, WI: American Society of Agronomy and Soil Science Society of America; 1982. pp. 595–624.

[26] Olsen SR, Cole CV, Watanabe FS, Dean LA. Estimation of available phosphorus in soils by extraction with sodium bicarbonate. Circular No. 939. Washington, DC: United States Department of Agriculture; 1954.

[27] Chapman HD. Cation-exchange capacity. In: Black CA, editor. Methods of Soil Analysis. Part 2: Chemical and Microbiological Properties. Madison, WI: American Society of Agronomy; 1965. pp. 891–901.

[28] Gomez KA, Gomez AA. Statistical Procedures for Agricultural Research. 2nd ed. New York: John Wiley & Sons; 1984.

[29] SAS Institute Inc. SAS/STAT 9.3 User’s Guide. Cary, NC: SAS Institute Inc.; 2013.

[30] Yin N, Geng N, Wang T, Wang H, Pan H, Yang Q, et al. Effect of acidification on clay minerals and surface properties of brown soil. Sustainability. 2022;15(1):179. doi: 10.3390/su15010179

[31] Baquy MA-A, Li J-Y, Jiang J, Mehmood K, Shi R-Y, Xu R-K. Critical pH and exchangeable Al of four acidic soils derived from different parent materials for maize crops. J Soils Sediments. 2017;18(4):1490–9. doi: 10.1007/s11368-017-1887-x

[32] Opala PA, Odendo M, Muyekho FN. Effects of lime and fertilizer on soil properties and maize yields in acid soils of Western Kenya. 2018.

[33] Landon JR. Booker Tropical Soil Manual: A Handbook for Soil Survey and Agricultural Land Evaluation in the Tropics and Subtropics. London: Routledge; 2014.

[34] Musinguzi P, Ebanyat P, Tenywa JS, Basamba TA, Tenywa MM, Mubiru DN. Critical soil organic carbon range for optimal crop response to mineral fertiliser nitrogen on a ferralsol. Ex Agric. 2016;52(4):635–53. doi: 10.1017/s0014479715000307

[35] Abera G, Wolde-Meskel E. Soil properties, and soil organic carbon stocks of tropical andosol under different land uses. J Soil Sci. 2013;03(03):153–62. doi: 10.4236/ojss.2013.33018

[36] Karltun E, Mamo T, Bekele T, Gameda S, Kidanu S. Towards Improved Fertilizer Recommendations in Ethiopia—Nutrient Indices for Categorization of Fertilizer Blends from EthioSIS Woreda Soil Inventory Data. Addis Ababa, Ethiopia: Ethiopian Agricultural Transformation Agency. 2013.

[37] Sapkota N, Airee S, Bhandari A, Mukherjee A. Integrated nutrient management in spring maize production in Nepal. Agrosyst Geosci Environ. 2025;8(3). doi: 10.1002/agg2.70178

[38] Yohannes Z, Yoseph T, Kiflu A, Ayalew T, Haile A. Improving maize productivity and food security through the application of organic amendments with liquid bio-slurry. Ukrainian J Ecol. 2024;14(1):6–25.

[39] Rahman MA, Rahman MM, Begum MF, Alam MF. Effect of bio compost, cow dung compost and NPK fertilizers on growth, yield and yield components of chili. Int J Biosci. 2012;2(1):51–5.

[40] Abdissa B, Kibebew K, Bobe B, Tesfaye B, Markku Y-H. Effects of lime, vermicompost and chemical P fertilizer on yield of maize in Ebantu District, Western highlands of Ethiopia. Afr J Agric Res. 2018;13(10):477–89. doi: 10.5897/ajar2017.12949

[41] Ibijola TO, Azeez JO, Adetunji MT, Oyekanmi AA. Effect of the sequential application of liquid organic manure and phosphorus on maize agronomic traits and P uptake in some tropical soils. J Plant Nutr. 2014;37(7):1040–55. doi: 10.1080/01904167.2014.881860

[42] Eleduma A, Aderibigbe A, Obabire S. Effect of cattle manure on the performances of maize (*Zea mays* L) grown in forest-savannah transition zone Southwest Nigeria. Int J Agric Sc Food Technol. 2020:110–4. doi: 10.17352/2455-815x.000063

[43] Singh S, Misal NB. Effect of different levels of organic and inorganic fertilizers on maize (*Zea mays* L.). IJARe. 2021;(Of). doi: 10.18805/ijare.a-5231

[44] Xu Y, Li Y, Yu Z, Jin J, Zhang C, Bao W, et al. Roles of soil organic acids and base cations in Mollisol acidification during the peak growth stage of crops. Soil Tillage Res. 2025;254:106726. doi: 10.1016/j.still.2025.106726

[45] Regasa A, Haile W, Abera G. Synergistic effects of lime, vermicompost, and phosphorus fertilizer enhance maize productivity and soil quality in acidic soils of Ethiopia. Discov Soil. 2026;3(1). doi: 10.1007/s44378-026-00199-9

[46] Habdollo D, Lulu M, Adinew A. Response of maize (Zea mays L.) to different rates of farmyard manure and lime in Angacha District, Kembata Zone, Central Ethiopia. Appl Environ Soil Sci. 2026;2026:4667174.

[47] Rajesh SR, Desai BK, Vinodakumar SN. Growth, yield and yield components of hybrid maize (Zea mays L.) as influenced by different nutrient combinations under integrated farming system. Trends Biosci. 2014;7(2):78–82.

[48] Kimiti WW, Mucheru-Muna MW, Mugwe JN, Ngetich KF, Kiboi MN, Mugendi DN. Lime, manure and inorganic fertilizer effects on soil chemical properties, maize yield and profitability in acidic soils in central highlands of Kenya. Asian J Environ Ecol. 2021;16(4):40–51. doi: 10.9734/ajee/2021/v16i330250

[49] Dhiman D, Sharma R, Sankhyan NK, Sepehya S, Sharma SK, Kumar R. Effect of regular application of fertilizers, manure and lime on soil health and productivity of wheat in an acid Alfisol. J Plant Nutr. 2019;42(19):2507–21. doi: 10.1080/01904167.2019.1659317

[50] Agegnehu G, Yirga C, Erkossa T. Soil Acidity Management. Addis Ababa, Ethiopia: Ethiopian Institute of Agricultural Research (EIAR); 2019.

[51] Bedadi B, Beyene S, Erkossa T, Fekadu E. Soil Management. In: Beyene S, Regassa A, Mishra BB, Haile M, editors. The Soils of Ethiopia. Cham: Springer International Publishing. 2023. pp. 193–234. doi: 10.1007/978-3-031-17012-6_9

[52] Howe JA, McDonald MD, Burke J, Robertson I, Coker H, Gentry TJ, et al. Influence of fertilizer and manure inputs on soil health: A review. Soil Security. 2024;16:100155. doi: 10.1016/j.soisec.2024.100155

[53] Sharma SP, Subehia SK. Effects of twenty-five years of fertilizer use on maize and wheat yields and quality of an acidic soil in the Western Himalayas. Ex Agric. 2003;39(1):55–64. doi: 10.1017/s0014479702001035

[54] Moges T, Melese A, Tadesse G. Effects of lime and phosphorus fertilizer levels on growth and yield components of malt barley (Hordeum distichum L.) in Angolelana Tera District, North Shewa Zone, Ethiopia. Adv Plants Agric Res. 2018;8(6):582–9. doi: 10.15406/apar.2018.08.00356

[55] Tekalign T, Haque I, Aduayi EA. Soil, Plant, Water, Fertilizer, Animal Manure and Compost Analysis. 13. Addis Ababa, Ethiopia: International Livestock Centre for Africa (ILCA). 1991. https://hdl.handle.net/10568/5064

[56] Mugwira LM, Nyamangara J, Hikwa D. Effects of manure and fertilizer on maize at a research station and in a smallholder (peasant) area of Zimbabwe. Commun Soil Sci Plant Anal. 2002;33(3–4):379–402. doi: 10.1081/css-120002752

[57] Agegnehu G, Bekele T. On-farm integrated soil fertility management in wheat on Nitisols of central Ethiopian highlands. Ethiop J Nat Resour. 2005;7(2):141–55.

[58] Venkatesh MS, Majumdar B, Kumar K. Effect of phosphorus, FYM and lime on yield, P uptake by maize and forms of soil acidity in Typic Hapludalf of Meghalaya. J Ind Soc Soil Sci. 2002;50(3):254–8.

[59] Aziz T, Ullah S, Sattar A, Nasim M, Farooq M, Khan MM. Nutrient availability and maize (Zea mays) growth in soil amended with organic manures. Int J Agric Biol. 2010;12(4):621–4.

[60] Minardi S, Harieni S, Anasrullah A, Purwanto H. Soil fertility status, nutrient uptake, and maize (*Zea mays* L.) yield following organic matters and P fertilizer application on andisol. IOP Conf Ser: Mater Sci Eng. 2017;193:012054. doi: 10.1088/1757-899x/193/1/012054

[61] Onwonga RN, Lelei JJ, Freyer B, Friedel JK, Mwonga SM, Wandhawa P. Low cost technologies for enhancing N and P availability and maize (*Zea mays* L.) performance on acid soils. World J Agric Sci. 2008;4(5):862–73.

[62] Ardestani MM, van Gestel CAM. Sorption and pH determine the long-term partitioning of cadmium in natural soils. Environ Sci Pollut Res Int. 2016;23(18):18492–501. doi: 10.1007/s11356-016-7034-1 27287493

[63] Agegnehu G, vanBeek C, Bird MI. Influence of integrated soil fertility management in wheat and tef productivity and soil chemical properties in the highland tropical environment. J Soil Sci Plant Nutr. 2014;(ahead):0–0. doi: 10.4067/s0718-95162014005000042

[64] Bayu T. Effect of integrated fertility management on soil physicochemical properties and yield and yield components of maize (*Zea mays* L.) and tef (Eragrostis tef) at Yilmana Densa District, Northwestern Ethiopia. Ethiopia: Bahir Dar University; 2017. pp. 116.

