# Supplementary material for: Maize (zea mays l.) yield, and selected chemical properties of soils as influenced by lime and manure application in acid soils of Meket District, northeastern Ethiopia
Source: PLoS One. 2026 Oct 12;21(10):e0360551. doi: 10.1371/journal.pone.0360551 (PMC13657311; doi:10.1371/journal.pone.0360551)
Supplement: Supplementary file 1 [file pone.0360551.s002.docx]

| **Obs** | **Rep** | **lime** | **manure** | **pH** | **SOC** | **TN** | **AVP** | **CEC** | **EXAC** |
| --- | --- | --- | --- | --- | --- | --- | --- | --- | --- |
| **1** | 1 | 1 | 1 | 4.89 | 1.25 | 0.11 | 7.27 | 21.40 | 2.21 |
| **2** | 2 | 1 | 1 | 5.01 | 1.26 | 0.12 | 7.34 | 23.20 | 1.82 |
| **3** | 3 | 1 | 1 | 4.91 | 1.25 | 0.10 | 8.01 | 23.50 | 2.01 |
| **4** | 1 | 1 | 2 | 4.98 | 1.32 | 0.13 | 8.24 | 24.20 | 1.78 |
| **5** | 2 | 1 | 2 | 5.02 | 1.34 | 1.14 | 8.54 | 23.80 | 1.69 |
| **6** | 3 | 1 | 2 | 5.11 | 1.36 | 0.15 | 8.97 | 23.90 | 1.54 |
| **7** | 1 | 1 | 3 | 5.12 | 1.45 | 0.14 | 8.97 | 28.51 | 1.79 |
| **8** | 2 | 1 | 3 | 5.21 | 1.52 | 0.19 | 8.78 | 28.95 | 1.81 |
| **9** | 3 | 1 | 3 | 5.13 | 1.58 | 0.21 | 9.02 | 29.41 | 1.82 |
| **10** | 1 | 1 | 4 | 5.24 | 1.59 | 0.24 | 9.25 | 28.78 | 1.54 |
| **11** | 2 | 1 | 4 | 5.35 | 1.72 | 0.21 | 9.37 | 29.89 | 1.59 |
| **12** | 3 | 1 | 4 | 5.45 | 1.89 | 0.24 | 9.87 | 29.20 | 1.42 |
| **13** | 1 | 2 | 1 | 5.31 | 1.29 | 0.12 | 9.36 | 23.20 | 1.75 |
| **14** | 2 | 2 | 1 | 5.42 | 1.27 | 0.13 | 9.21 | 21.80 | 1.52 |
| **15** | 3 | 2 | 1 | 5.41 | 1.24 | 0.12 | 9.27 | 23.80 | 1.50 |
| **16** | 1 | 2 | 2 | 5.48 | 1.32 | 0.15 | 9.56 | 24.63 | 1.42 |
| **17** | 2 | 2 | 2 | 5.51 | 1.39 | 0.18 | 10.23 | 26.87 | 1.46 |
| **18** | 3 | 2 | 2 | 5.49 | 1.46 | 0.18 | 11.35 | 27.23 | 1.48 |
| **19** | 1 | 2 | 3 | 5.51 | 1.90 | 0.19 | 10.87 | 27.85 | 1.47 |
| **20** | 2 | 2 | 3 | 5.49 | 1.98 | 0.18 | 11.23 | 28.64 | 1.46 |
| **21** | 3 | 2 | 3 | 5.52 | 1.85 | 0.21 | 11.32 | 29.52 | 1.41 |
| **22** | 1 | 2 | 4 | 5.46 | 1.98 | 0.21 | 11.58 | 29.87 | 1.32 |
| **23** | 2 | 2 | 4 | 5.53 | 2.32 | 0.22 | 11.34 | 28.96 | 1.20 |
| **24** | 3 | 2 | 4 | 5.57 | 2.01 | 0.21 | 11.52 | 27.96 | 1.25 |
| **25** | 1 | 3 | 1 | 5.61 | 1.27 | 0.12 | 10.32 | 23.28 | 1.18 |
| **26** | 2 | 3 | 1 | 5.69 | 1.29 | 0.13 | 10.63 | 24.68 | 1.16 |
| **27** | 3 | 3 | 1 | 5.47 | 1.30 | 0.12 | 10.71 | 25.64 | 0.92 |
| **28** | 1 | 3 | 2 | 5.67 | 1.78 | 0.15 | 11.23 | 26.89 | 0.96 |
| **29** | 2 | 3 | 2 | 5.61 | 1.82 | 0.14 | 11.45 | 26.86 | 0.91 |
| **30** | 3 | 3 | 2 | 5.63 | 1.78 | 0.14 | 11.41 | 27.64 | 0.98 |
| **31** | 1 | 3 | 3 | 5.61 | 1.85 | 0.18 | 11.58 | 28.91 | 0.97 |
| **32** | 2 | 3 | 3 | 5.60 | 1.85 | 0.17 | 12.35 | 27.61 | 0.92 |
| **33** | 3 | 3 | 3 | 5.62 | 1.93 | 0.18 | 13.58 | 27.68 | 0.94 |
| **34** | 1 | 3 | 4 | 5.67 | 1.98 | 0.21 | 14.21 | 29.10 | 0.90 |
| **35** | 2 | 3 | 4 | 5.72 | 2.07 | 0.21 | 14.65 | 28.97 | 0.85 |
| **36** | 3 | 3 | 4 | 5.81 | 2.09 | 0.24 | 14.37 | 29.87 | 0.70 |
