# Supplementary material for: Maize (zea mays l.) yield, and selected chemical properties of soils as influenced by lime and manure application in acid soils of Meket District, northeastern Ethiopia
Source: PLoS One. 2026 Oct 12;21(10):e0360551. doi: 10.1371/journal.pone.0360551 (PMC13657311; doi:10.1371/journal.pone.0360551)
Supplement: Supplementary file 2 [file pone.0360551.s003.docx]

Yield data

| **Obs** | **Rep** | **lime** | **manure** | **AGDB** | **GY** | **SY** |
| --- | --- | --- | --- | --- | --- | --- |
| **1** | 1 | 1 | 1 | 5514 | 1945 | 3569 |
| **2** | 2 | 1 | 1 | 5996 | 2101 | 3895 |
| **3** | 3 | 1 | 1 | 5710 | 2021 | 3689 |
| **4** | 1 | 1 | 2 | 8154 | 3565 | 4589 |
| **5** | 2 | 1 | 2 | 7104 | 3015 | 4089 |
| **6** | 3 | 1 | 2 | 7187 | 2936 | 4251 |
| **7** | 1 | 1 | 3 | 10133 | 3223 | 6910 |
| **8** | 2 | 1 | 3 | 11733 | 3823 | 7910 |
| **9** | 3 | 1 | 3 | 12350 | 3454 | 8896 |
| **10** | 1 | 1 | 4 | 11998 | 3844 | 8154 |
| **11** | 2 | 1 | 4 | 12137 | 4123 | 8014 |
| **12** | 3 | 1 | 4 | 13770 | 3985 | 9785 |
| **13** | 1 | 2 | 1 | 6512 | 2025 | 4487 |
| **14** | 2 | 2 | 1 | 6512 | 2025 | 4487 |
| **15** | 3 | 2 | 1 | 7170 | 2532 | 4638 |
| **16** | 1 | 2 | 2 | 9816 | 3594 | 6222 |
| **17** | 2 | 2 | 2 | 9823 | 2872 | 6951 |
| **18** | 3 | 2 | 2 | 9858 | 3489 | 6369 |
| **19** | 1 | 2 | 3 | 11405 | 4295 | 7110 |
| **20** | 2 | 2 | 3 | 11215 | 3968 | 7247 |
| **21** | 3 | 2 | 3 | 11091 | 3998 | 7093 |
| **22** | 1 | 2 | 4 | 13228 | 3565 | 9663 |
| **23** | 2 | 2 | 4 | 15111 | 3896 | 11215 |
| **24** | 3 | 2 | 4 | 16072 | 3485 | 12587 |
| **25** | 1 | 3 | 1 | 11783 | 3196 | 8587 |
| **26** | 2 | 3 | 1 | 11067 | 3487 | 7580 |
| **27** | 3 | 3 | 1 | 10754 | 3269 | 7485 |
| **28** | 1 | 3 | 2 | 12082 | 3586 | 8496 |
| **29** | 2 | 3 | 2 | 12153 | 3784 | 8369 |
| **30** | 3 | 3 | 2 | 12260 | 3263 | 8997 |
| **31** | 1 | 3 | 3 | 11795 | 4110 | 7685 |
| **32** | 2 | 3 | 3 | 12048 | 3925 | 8123 |
| **33** | 3 | 3 | 3 | 11126 | 3985 | 7141 |
| **34** | 1 | 3 | 4 | 13570 | 3785 | 9785 |
| **35** | 2 | 3 | 4 | 13574 | 3987 | 9587 |
| **36** | 3 | 3 | 4 | 13456 | 3985 | 9471 |
